# Enhanced Production of (*R*)-3-Hydroxybutyrate Oligomers by Coexpression of Molecular Chaperones in Recombinant *Escherichia coli* Harboring a Polyhydroxyalkanoate Synthase Derived from *Bacillus cereus* YB-4

**DOI:** 10.3390/microorganisms10020458

**Published:** 2022-02-16

**Authors:** Saki Goto, Yuki Miyahara, Seiichi Taguchi, Takeharu Tsuge, Ayaka Hiroe

**Affiliations:** 1 Department of Chemistry for Life Sciences and Agriculture, Faculty of Life Sciences, Tokyo University of Agriculture, 1-1-1 Sakuragaoka, Setagaya, Tokyo 156-8502, Japan; sg207296@nodai.ac.jp (S.G.); st206172@nodai.ac.jp (S.T.); 2 MIRAI, Japan Science and Technology Agency (JST), 4-1-8 Honcho, Kawaguchi, Saitama 332-0012, Japan; miyahara.y.aa@m.titech.ac.jp (Y.M.); tsuge.t.aa@m.titech.ac.jp (T.T.); 3 Department of Materials Science and Engineering, School of Materials and Chemical Technology, Tokyo Institute of Technology, 4259 Nagatsuta, Midori-ku, Yokohama 226-8502, Japan

**Keywords:** oligomer, polyhydroxyalkanoate (PHA), PHA synthase, secretory production, chaperones

## Abstract

The biodegradable polyester poly-(*R*)-3-hydroxybutyrate [P(3HB)] is synthesized by a polymerizing enzyme called polyhydroxyalkanoate (PHA) synthase and accumulates in a wide variety of bacterial cells. Recently, we demonstrated the secretory production of a (*R*)-3HB oligomer (3HBO), a low-molecular-weight P(3HB), by using recombinant *Escherichia coli* expressing PHA synthases. The 3HBO has potential value as an antibacterial substance and as a building block for various polymers. In this study, to construct an efficient 3HBO production system, the coexpression of molecular chaperones and a PHA synthase derived from *Bacillus cereus* YB-4 (PhaRC_YB4_) was examined. First, genes encoding enzymes related to 3HBO biosynthesis (*phaRC_YB4_*, *phaA* and *phaB* derived from *Ralstonia eutropha* H16) and two types of molecular chaperones (*groEL*, *groES*, and *tig*) were introduced into the *E. coli* strains BW25113 and BW25113Δ*adhE*. As a result, coexpression of the chaperones promoted the enzyme activity of PHA synthase (approximately 2–3-fold) and 3HBO production (approximately 2-fold). The expression assay of each chaperone and PHA synthase subunit (PhaR_YB4_ and PhaC_YB4_) indicated that the combination of the two chaperone systems (GroEL-GroES and TF) supported the folding of PhaR_YB4_ and PhaC_YB4_. These results suggest that the utilization of chaperone proteins is a valuable approach to enhance the formation of active PHA synthase and the productivity of 3HBO.

## 1. Introduction

Polyhydroxyalkanoates (PHAs) are biodegradable plastics that are synthesized by various microorganisms [1,2,3]. Depending on monomer structure, PHAs can be categorized into three groups: short-chain-length PHAs (SCL-PHAs), containing 3–5 carbon atoms in the monomer; medium-chain-length PHAs (MCL-PHAs), containing 6–14 carbon atoms in the monomer; and SCL-MCL-PHAs, containing both SCL and MCL monomer units. PHA synthase is a key enzyme in the polymerization of PHA. PHA synthases can be divided into four classes according to their substrate specificities and subunit compositions [4]. Whereas class I and II PHA synthases are composed of single subunits of PhaC, class III and IV PHA synthases are composed of two heterosubunits, with PhaE and PhaC forming PhaEC and PhaR, and PhaC forming PhaRC [5]. Regarding substrate specificity, class I, III, and IV PHA synthases prefer SCL monomers as substrates, whereas class II PHA synthases are specific to MCL monomers. Attempts have been made to synthesize PHAs with favorable properties using various natural and engineered PHA synthases [6,7,8]. The homopolymer poly[(*R*)-3-hydroxybutyrate] [P(3HB)], the most common SCL-PHA, is stiff and brittle, which limits its range of applications. P(3HB-*co*-3-hydroxyhexianoate) [P(3HB-*co*-3HHx)] [9]; P(3HB-*co*-3-hydroxyalkanoate), consisting of 3HA units of C_6_–C_12_ [10]; P(D-lactate-*co*-3HB) [P(LA-*co*-3HB)], a D-lactate-based polymer [11]; and MCL-PHA homopolymers [12] have improved properties, exhibiting transparency and flexibility.

In recent studies, the secretory production of low-molecular-weight D-lactate-based polymers and P(3HB) polymers, referred to as D-lactate-based oligomers (D-LAOs) and 3HB oligomers (3HBOs), respectively, was demonstrated using *Escherichia coli* heterologously expressing PHA synthases [13,14,15,16,17,18]. Unlike polymer production in cells, secretory production is a continuous process because of the lack of a cell volume limitation. In particular, 3HBOs are found in nature, for example, as a spider sex pheromone and a growth-promoting factor for bacteria [19,20,21,22]. Recently, the antibacterial activity of a 3HBO and an application of this oligomer (as a textile fiber) were also reported [21,23]. 3HBOs are expected to have utility as bioactive compounds and macromonomers for various polymers, such as polyurethanes [17,24].

Our group first developed an artificial 3HBO production system using recombinant *E. coli*. The recombinant *E. coli* strain had a gene cassette consisting of the β-ketothiolase (*phaA*) gene, the NADPH-dependent acetoacetyl-CoA reductase gene (*phaB*), and the PHA synthase gene (*phaC*). In particular, PHA synthases derived from *Bacillus cereus* YB-4 (PhaRC_YB4_) and from *Aeromonas caviae* (PhaC_Ac_) were superior in terms of 3HBO productivity [16]. To date, 3HBO generation has been observed in the presence of host-produced (endogenous) and/or supplemented (exogeneous) alcohols. This phenomenon indicates that i) alcohols are frequently added to the carboxy terminus of oligomers in the polymerization process via PHA synthases and lead to the generation of new oligomer chains (termed the chain transfer reaction) and ii) alcohols degrade elongated polymer chains via specific PHA synthases such as PhaRC_YB4_ under monomer-depleted conditions in the late phase of cultivation (termed the alcoholysis reaction) [13,16]. In the process of alcoholysis, since intracellular P(3HB) is gradually degraded, a decrease in the molecular weight of P(3HB) and the generation of 3HBOs are simultaneously observed [25,26]. Through these two reactions, the secretory production of 3HBO end-capped with host-produced ethanol [16] and 3HBO end-capped with supplemented diethylene glycol (DEG) [17] has been reported.

In this study, we focused on the expression level of PHA synthase to establish an efficient 3HBO production system. In a previous study of P(3HB) (polymer) production, when PHA synthase was highly expressed, an increase in the polymer amount was observed because of an increase in the polymerization starting point [27,28]. In parallel, higher expression of PHA synthase led to a decrease in the molecular weight of P(3HB) due to the competition for the 3HB monomer by additional PHA synthases. With regard to the 3HBO production system, we predicted that higher expression of PHA synthase would enhance the 3HBO amount based on the same principle. To improve the expression level of PHA synthase, we attempted to utilize the major molecular chaperones in *E. coli*. Generally, a majority of proteins must be correctly folded into specific three-dimensional shapes for functionalization. However, nascent polypeptide chains are prone to misfolding and aggregation. Molecular chaperones prevent protein misfolding and assist in the refolding of misfolded proteins. Previous studies have shown that PHA synthases easily form inclusion bodies when overexpressed in *E. coli* and that the use of molecular chaperones is effective for the formation of active PHA synthases [29]. One of the major chaperones, trigger factor (TF), binds to the bacterial ribosome and interacts with emerging nascent polypeptide chains [30,31]. Another chaperone, GroEL, and its cofactor GroES, promote protein folding by sequestering nonnative polypeptides in a cage-like structure [32,33]. Here, we report the effect of chaperone coexpression on 3HBO production.

## 2. Materials and Methods

### 2.1. Bacterial Strains and Plasmids

The bacterial strains and plasmids used in this study are listed in Table 1. pGEM-*phaRC*_YB4_*AB* [34] was introduced into *E. coli* BW25113 or its mutant strain lacking the alcohol dehydrogenase gene (BW25113Δ*adhE*) for 3HBO or P(3HB) production. The chaperone plasmid pG-Tf2 containing GroEL-GroES/TF genes (*groEL*, *groES*, and *tig*) was selected from the Chaperone Plasmid Set (Takara Bio Inc., Shiga, Japan) according to a previous study [29]. In pG-Tf2, the *groEL*, *groES*, and *tig* genes are located downstream of the Pzt-1 (tet) promoter and can be expressed by induction of tetracycline. Another chaperone plasmid, pG-KJE8, containing the *groEL* and *groES* genes, was also used in subsequent assays. pET15b-*phaR*_YB4_ or pET15b-*phaC*_YB4_ [25] was introduced into *E. coli* BL21(DE3) for the expression of PhaR_YB4_ or PhaC_YB4_, respectively. To maintain the plasmids within the cells, 100 mg/L ampicillin (for pGEM-*phaRC*_YB4_*AB*), 20 mg/L chloramphenicol (for pG-KJE8 and pG-Tf2), and 50 mg/L carbenicillin (for pET15b-*phaR*_YB4_ and pET15b-*phaC*_YB4_) were added to the medium as appropriate.

### 2.2. Culture Conditions for 3HBO Production

For 3HBO production, four strains, namely, *E. coli* BW25113 or BW25113Δ*adhE* harboring pGEM-*phaRC*_YB4_*AB* or pGEM-*phaRC*_YB4_*AB* + pG-Tf2, were prepared. Each transformant was cultivated in 1.7 mL of Luria–Bertani (LB) medium (10 g/L NaCl, 10 g/L tryptone, and 5 g/L yeast extract) containing the appropriate antibiotics at 30 °C for 16 h as a preculture. One milliliter of the preculture was inoculated into a 500 mL apple-shaped flask with 100 mL of LB medium containing glucose (20 g/L) and the appropriate antibiotics. For strains harboring pG-Tf2, tetracycline (5 µg/L) was added as an inducer for chaperones at the beginning of cultivation. Cells were cultivated at 30 °C for 48 h (130 strokes/min), harvested by centrifugation (10,000× *g*, 10 min, 24 °C), washed twice with pure water and lyophilized. The culture supernatant was collected for measuring the 3HBO and 3HB levels.

### 2.3. Quantification of Extracellular 3HBOs/3HB, Extracellular Ethanol, Intracellular 3HBOs and P(3HB)

The concentrations of extracellular 3HBOs/3HB and intracellular 3HBOs were determined by an enzyme assay as described previously [16,17]. In the same way, the concentration of extracellular ethanol was measured by an enzyme assay using F-kit Ethanol (J. K. International, Tokyo, Japan). The total amounts of P(3HB) and intracellular 3HBOs were determined by gas chromatography (GC) using a GC2030 instrument (Shimadzu, Kyoto, Japan) equipped with a flame ionization detector (FID). Approximately 15 mg of lyophilized cells was methanolized with 15% (*v/v*) sulfuric acid as previously described [36]. The amount of P(3HB) was calculated by subtracting the amount of intracellular 3HBOs determined by the above enzyme assay.

### 2.4. Extraction of 3HBOs and ESI-TOF-MS Analysis

For the assay of the molecular weight (polymerization degree) of 3HBOs, extracellular 3HBOs were extracted from cell-free (filtered) culture supernatants. First, the cell-free supernatant was added to an equivalent volume of chloroform, and the mixture was stirred for at least 1 h. After cessation of stirring, the mixture was allowed to stand until two layers were clearly observed. The chloroform layer was recovered, and an equivalent volume of pure water was added to and mixed with the extract to remove the medium components and 3HB monomer. This washing step using pure water was repeated twice. Then, the recovered chloroform layer was dried to obtain an extracellular 3HBO sample. Intracellular 3HBOs were extracted from the dried cells by soaking in methanol. After forceful centrifugation, the cell debris was removed by a polytetrafluoroethylene (PTFE) filter, and intracellular 3HBO samples were obtained. The 3HBO samples were dissolved in methanol and filtered before being subjected to electrospray ionization-time of flight-mass spectrometry (ESI-TOF-MS) analysis.

### 2.5. Purification of P(3HB) and GPC Analysis

The P(3HB) accumulated in the cells was extracted with chloroform and purified by reprecipitation with methanol as described previously [16]. The molecular weights (*M*_n_ and *M*_w_) and molecular weight distribution (*M*_w_/*M*_n_) were determined by gel permeation chromatography (GPC). GPC measurements were performed at 40 °C using a Shimadzu 20A GPC system and a 20A refractive index detector (Shimadzu, Kyoto, Japan) equipped with a Shimadzu GPC-80MC column joined to two Shodex K806M columns (Showa Denko KK, Tokyo, Japan). Chloroform was used as the mobile phase at a flow rate of 0.8 mL/min. Samples for GPC analysis were prepared at a P(3HB) concentration of 1.0 mg/mL and passed through a 0.2-μm PTFE filter. Then, 100 µL of each sample was injected into the GPC system. Low-polydispersity polystyrene standards were used to generate a calibration curve, and the relative molecular weight of P(3HB) was calculated based on the calibration curve.

### 2.6. PHA Synthase Activity Assay

To measure the activity of PHA synthase during the production of 3HBOs, cells at 24 h of cultivation were collected, washed with 50 mM NaPi buffer, and stored at −80 °C until the activity assay. The cells were resuspended in 50 mM NaPi buffer, disrupted by sonication (10 times × 30 s), and centrifuged at 1500× *g* for 5 min at 4 °C to obtain crude extract containing PHA synthase, which was bound to PHA granules. The activity of PHA synthase was assayed by measuring the decrease in absorbance at 236 nm (ε = 4500 [M^−1^cm^−1^]) caused by cleavage of the thioester bond of (*R*)-3HB-CoA [37]. The reaction was initiated by the addition of 20 µg of crude protein into the reaction buffer, consisting of 50 mM NaPi buffer (pH 7.0) and (*R*)-3HB-CoA (100 µM). The specific activity (U/mg-protein) of PHA synthase was determined using the maximum velocities of each reaction. (*R*)-3HB-CoA was prepared as described previously [16].

### 2.7. Expression of PhaR_YB4_ and PhaC_YB4_ with Molecular Chaperones

To evaluate the effect of molecular chaperones on the expression of PhaR_YB4_ and PhaC_YB4_, six transformants were prepared by transforming *E. coli* BL21(DE3) with pET15b-*phaR*_YB4_, pET15b-*phaR*_YB4_+pG-KJE8 (encoding the GroEL-GroES genes), pET15b-*phaR*_YB4_+pG-Tf2 (encoding the GroEL-GroES and TF genes), pET15b-*phaC*_YB4_, pET15b-*phaC*_YB4_+pG-KJE8 or pET15b-*phaC*_YB4_+pG-Tf2. The resulting six transformants were cultivated in 1.7 mL of LB medium. The preculture was inoculated into a 500 mL apple-shaped flask with 100 mL of LB medium with antibiotics. To generate chaperone proteins before PhaR_YB4_ and PhaC_YB4_ expression, the inducer for chaperones, tetracycline (5 or 10 µg/L), was added to the medium at the beginning of cultivation. Cells were cultivated with shaking (130 strokes/min) at 30 °C until the optical density at 600 nm (OD_600_) reached 0.4–0.6. Then, 100 µL of 0.1 M isopropyl-β-D-1-thiogalactopyranoside (IPTG) was added to the medium (final concentration of 0.1 mM), and the culture was grown at 20 °C for 8 h with shaking. The cells were harvested, washed with 50 mM sodium phosphate buffer (NaPi, pH 7.0), and stored at −80 °C. Subsequently, the cells were resuspended in the same buffer, disrupted by sonication (10 times × 30 s), and centrifuged at 20,400× *g* for 20 min at 4 °C. The supernatant (soluble protein fraction) and the pellet (insoluble fraction) were resuspended in 50 mM NaPi buffer, and the protein concentration in each fraction was determined using a Qubit™ Protein Assay Kit (Invitrogen, Carlsbad, CA, USA) according to the manufacturer’s instructions. The expression of PhaR_YB4_ and PhaC_YB4_ was confirmed by sodium dodecyl sulfate–polyacrylamide gel electrophoresis (SDS–PAGE) using 10 and 12.5 wt% gels, respectively. Ten micrograms of total protein was loaded per sample, and the gels were stained with Coomassie Brilliant Blue (CBB). The expression levels of PhaR_YB4_ and PhaC_YB4_ were determined using ImageJ software (https://imagej.nih.gov/ij/index.html (accessed on 27 January 2022)).

## 3. Results

### 3.1. Effect of Chaperones on 3HBO Production

To examine the effect of chaperones on 3HBO production, *E. coli* BW25113 harboring the plasmid pGEM-*phaRC*_YB4_*AB* alone or the plasmid pair pGEM-*phaRC*_YB4_*AB*+pG-Tf2 was grown in LB medium containing 20 g/L glucose (Table 2). As shown in Table 2, the total residual cell mass (RCM) and 3HB level (intracellular P(3HB) + intracellular 3HBOs + extracellular 3HBOs + extracellular 3HB) in the control strain (10.08 ± 0.06 g/L) and chaperone-expressing strain (10.07 ± 0.03 g/L) were equal. By comparison, the total amount of 3HBOs (extracellular and intracellular) increased 1.8-fold upon coexpression of the GroEL-GroES/TF chaperones (control, 0.92 ± 0.05 g/L; +pG-Tf2, 1.75 ± 0.08 g/L), and the amount of P(3HB) decreased from 7.41 ± 0.09 g/L to 6.08 ± 0.15 g/L. In addition, the molecular weight (*M*_n_ and *M*_w_) of P(3HB) isolated from the strain coexpressing chaperones was lower than that in the control strain, as previously reported [16,29]. The polydispersity (defined as the ratio of *M*_w_ to *M*_n_) was not changed by the expression of chaperones (Table 2, Figure S1A).

**Figure 1:**
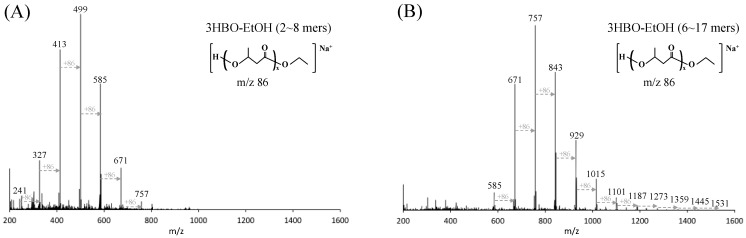
ESI-TOF-MS charts of extracellular (**A**) and intracellular (**B**) 3HBOs. 3HBOs were purified from the culture supernatant or cells of *E. coli* BW25113 harboring pGEM-*phaRC*_YB4_*AB* + pG-Tf2 after 48 h of cultivation. ESI-TOF-MS was performed in positive mode with an applied voltage of 120 V.

The ESI-TOF-MS charts (Figure 1) show the molecular weights of the extracellular and intracellular 3HBOs produced by the chaperone-expressing strain. The molecular weights of extracellular (Figure 1A) and intracellular 3HBOs (Figure 1B) were 200–800 (2–8 mers) and 500–1600 (6–17 mers), respectively. The peak interval, *m*/*z* 86, indicates a repeating 3HB unit, and the *m*/*z* of each peak matches that of sodium-adducted 3HBOs end-capped with ethanol, as previously reported [16,17]. As shown in Figure 1, the peaks for the extracellular and intracellular 3HBOs corresponded to 5 and 8 mers, respectively, and the spectra were the same as those of the strain without chaperones (data not shown).

**Figure 2:**
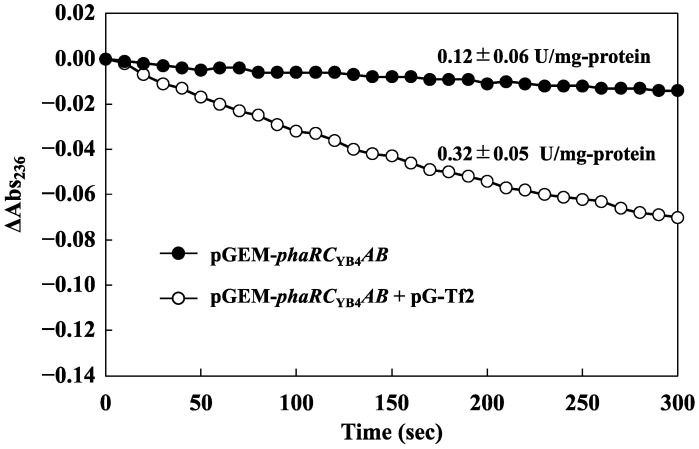
Specific activity of PhaRC_YB4_ with/without the chaperone plasmid pG-Tf2. Specific activities were determined by using the change in absorbance at 236 nm at maximum velocity.

To investigate the relationship between the increased yield of 3HBOs and the proportion of the active form of PHA synthase, an in vitro enzyme assay was performed. The enzyme activity of the chaperone-expressing strain was 3.0-fold higher than that of the control strain (Figure 2). SDS–PAGE analysis also showed a higher abundance of PhaR_YB4_ in the coexpression strain (data not shown). By comparison, the ethanol concentration in the culture medium was 1.5-fold higher than that in the chaperone-nonexpressing strain (control, 0.46 ± 0.02 g/L; +pG-Tf2, 0.68 ± 0.02 g/L).

### 3.2. Time Course Pattern of 3HBO Production with Coexpression of Molecular Chaperones

**Figure 3:**
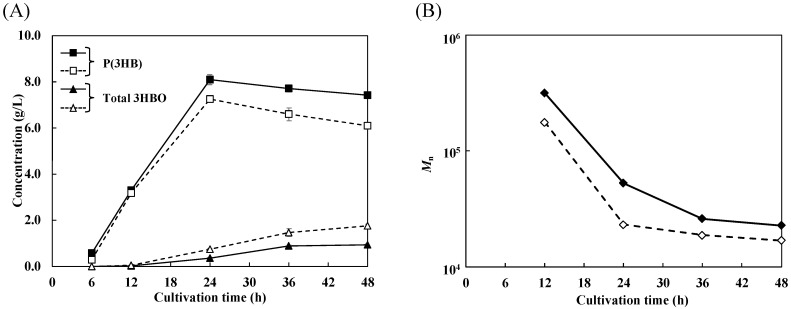
Time course data for the cultivation of recombinant *E. coli* BW25113 harboring pGEM-*phaRC*_YB4_*AB* (solid line) or pGEM-*phaRC*_YB4_*AB* + pG-Tf2 (broken line). (**A**) Concentrations of P(3HB) and total 3HBOs (intracellular 3HBOs + extracellular 3HBOs). (**B**) Number-average molecular weight of P(3HB) (12–48 h of cultivation). Purified P(3HB) at 6 h of cultivation could not be analyzed because it was present in only trace amount.

To trace the cultivation of the strain with or without chaperones, time course data were collected (Figure 3). 3HBO production in the two strains increased with culture time (Figure 3A). By comparison, P(3HB) production peaked at 24 h (Control, 8.09 g/L; +pG-Tf2, 7.23 g/L) and decreased during the 24–48 h phase. This phenomenon suggests that the produced polymer was converted to an oligomer by an alcoholysis reaction in the later phase of cultivation, as reported previously [17]. By comparing the two strains, it was confirmed that 3HBO production in the chaperone-expressing strain was higher than that in the control strain at all time points. This result suggests that the higher expression of PHA synthase in the chaperone-expressing strain enhanced both the chain transfer reaction (mainly in the growth phase) and the alcoholysis reaction (mainly in the stationary phase) and generated many more oligomers. The molecular weight of P(3HB) decreased with culture time (Figure 3B). As described in the Introduction section, when the expression of PHA synthase is higher, the molecular weight of P(3HB) is lower due to competition for 3HB monomers. Therefore, the molecular weight for the chaperone-expressing strain with more PHA synthases was relatively low at the beginning of cultivation and further decreased by the alcoholysis reaction.

### 3.3. Production of 3HBOs in Non-Ethanol-Producing E. coli with Coexpression of Molecular Chaperones

To clearly define the effect of high expression of PHA synthase on 3HBO production, host-produced ethanol, which functions as a substrate for chain transfer reactions and alcoholysis reactions, was removed from the culture using the non-ethanol-producing *E. coli* strain BW25113Δ*adhE.* As shown in Table 3, when the Δ*adhE* strain was used, the production level of 3HBOs was reduced by approximately 1/5 compared to that in the WT strain (Table 2), indicating that chain transfer and alcoholysis reactions using host-produced ethanol were repressed, as expected. The ethanol concentration in the culture supernatant was at trace levels regardless of the presence of chaperones. The total amount of 3HBOs from the Δ*adhE* strains increased 2.0-fold when the chaperones were expressed (Control, 0.18 ± 0.01 g/L; +pG-Tf2, 0.38 ± 0.02 g/L). In addition, the activity assay of the Δ*adhE* strains showed that the expression level of PHA synthase increased 2.3-fold upon chaperone expression (Control, 0.19 ± 0.05 U/mg-protein; +pG-Tf2, 0.44 ± 0.10 U/mg-protein). The molecular weight (*M*_n_ and *M*_w_) of P(3HB) isolated from the chaperone-expressing Δ*adhE* strain was lower than that in the nonexpressing Δ*adhE* strain and the chaperone-expressing/nonexpressing WT strains. By comparison, in the case of Δ*adhE* strains, the bimodal distribution of the P(3HB) molecular weight was confirmed (Figure S1B), suggesting that the trace level of ethanol produced by the Δ*adhE* strains acted as the substrate for chain transfer and alcoholysis reactions.

### 3.4. Effect of Chaperones on PhaR_YB4_ and PhaC_YB4_ Expression

**Figure 4:**
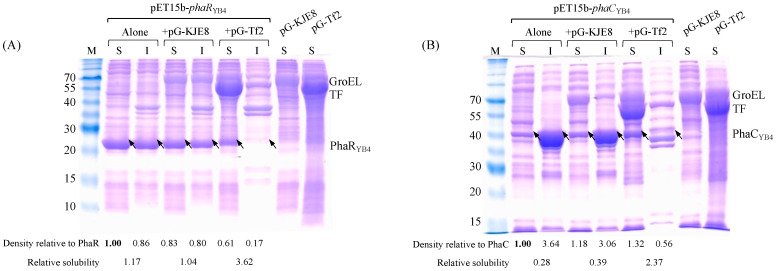
Effect of the chaperone plasmids pG-KJE8 and pG-Tf2 on the solubilization of PhaR_YB4_ and PhaC_YB4_. The levels of solubilized PhaR_YB4_ and PhaC_YB4_ were evaluated by SDS–PAGE. (**A**) *E. coli* BL21(DE3) harboring pET15b-*phaR*_YB4_, pET15b-*phaR*_YB4_ + pG-KJE8, pET15b-*phaR*_YB4_ + pG-Tf2, pG-KJE8, or pG-Tf2 or (**B**) *E. coli* BL21(DE3) harboring pET15b-*phaC*_YB4_, pET15b-*phaC*_YB4_ + pG-KJE8, pET15b-*phaC*_YB4_ + pG-Tf2, pG-KJE8, or pG-Tf2 was cultivated. For strains harboring the chaperone plasmids, 10 μg/L tetracycline (Tc) was added to induce the expression of the chaperones. After sonication of each cell sample, soluble (S) and insoluble (I) fractions were analyzed by SDS–PAGE. The arrows indicate the bands corresponding to PhaR_YB4_ and PhaC_YB4_. The relative densities of the bands were calculated using ImageJ software. The density relative to the band of PhaR_YB4_ or PhaC_YB4_ is the ratio of the density of each band to that of the band for the soluble fraction of PhaR_YB4_ or PhaC_YB4_ alone (bold). The relative solubilities are the ratios of the S to I fractions within each sample.

To determine the expression levels of PhaR_YB4_ and PhaC_YB4_ with/without chaperones, soluble and insoluble fractions were analyzed by SDS–PAGE (Figure 4). The predicted protein sizes of GroEL and TF were 60 and 56 kDa, respectively, and the corresponding bands were detected, as shown in Figure 4. However, GroES (10 kDa) could not be clearly identified. For both PhaR_YB4_ (18.5 kDa) and PhaC_YB4_ (41.7 kDa), the molecular weights were higher than those in a previous study [38], probably due to the N-terminal (His)_6_ tag. As shown in Figure 4A, although the GroEL-GroES chaperone did not change the solubilization level of PhaR_YB4_, GroEL-GroES/TF led to a 3.0-fold increase in PhaR_YB4_ solubility (PhaR_YB4_ alone, 1.17; +pG-pKJE8, 1.04; +pG-Tf2, 3.62). With regard to the other subunit of PhaC_YB4_, most of the PhaC_YB4_ was found in the insoluble fraction (Figure 4B), indicating that PhaC_YB4_ aggregated easily. Similar to the result for PhaR_YB4_, coexpression of the GroEL-GroES chaperones led to no change in PhaC_YB4_ solubility, but that of the GroEL-GroES/TF chaperones led to an 8.5-fold increase in PhaC_YB4_ solubility (PhaC_YB4_ alone, 0.28; +pG-pKJE8, 0.39; +pG-Tf2, 2.37). These results indicated that not the GroEL-GroES but the GroEL-GroES/TF chaperones support the folding of both PhaR_YB4_ and PhaC_YB4_. When the same experiments were performed with different concentrations of tetracycline (inducer of pG-Tf2), ranging from 0 to 10 µg/L, the bands of GroEL and TF were clearly detected with increasing tetracycline concentration (Figure S2).

## 4. Discussion

**Figure 5:**
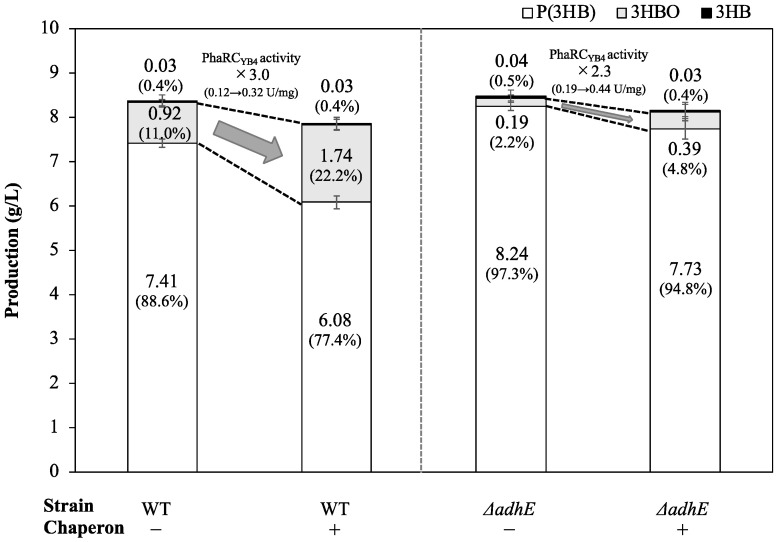
Production profile of 3HB compounds (P(3HB), 3HBO, and 3HB) and the distributional change caused by chaperone (GroEL-GroES/TF) expression. The data were derived from Table 2 and Table 3.

In this study, we investigated the relationship between the expression level of PHA synthase and 3HBO generation for the development of an efficient 3HBO production system. To enhance the expression level of PHA synthase (PhaRC_YB4_), the major chaperone molecules of *E. coli*, namely, GroEL-GroES and TF, were coexpressed with related enzymes for 3HBO synthesis. In the case of the first cultivation of the *E. coli* BW25113 strain, chaperon expression resulted in higher PHA synthase activity (3.0-fold) and higher 3HBO production (1.9-fold) (Figure 5, left side). In addition, the time course data of the cultivation showed higher 3HBO production in both the growth phase and later phase of cultivation (Figure 3), indicating a higher frequency of chain transfer reactions and alcoholysis reactions by the increase in the number of PHA synthase (PhaRC_YB4_). It was also revealed that the increased amount of PHA synthase led to a greater number of oligomer chains but did not affect the range of the polymerization degree of the oligomers. The generated 3HBOs represented an adductive form with ethanol at the carboxyl terminus (Figure 1) because endogenous ethanol functioned as a substrate for chain transfer reaction and alcoholysis reaction.

To remove the effect of ethanol on 3HBO production and evaluate the effect of the expression level of PHA synthase itself, subsequent cultivation was performed using the non-ethanol-producing strain (BW25113Δ*adhE*) as a host strain. As is the case with the wild-type strain, the expression levels of PHA synthase and 3HBO production were both increased (2.8- and 2.0-fold) by chaperone expression (Figure 5, right side). Chemical structure analysis (ESI-TOFM-MS) of 3HBOs from the Δ*adhE* strain revealed major amounts of the non-end-capped form of 3HBOs, and minor amounts of the ethanol end-capped form of 3HBOs which was derived from the trace level of ethanol produced by the Δ*adhE* strain (data not shown). To exclude the possibility that the slight difference in ethanol level affected the 3HBO amount, additional cultivation using Δ*adhE* strains was performed with an equal amount of exogenous ethanol (0.5, 1, 3, 5 and 10 g/L). As a result of cultivation (Table S1), 3HBO production was consistently higher (1.1–4.3-fold) under the chaperone expression conditions, indicating that not the ethanol level but the increase in PHA synthase itself enhanced 3HBO generation. Compared to the ethanol-producing strain (WT strain), the amount of 3HBOs was greatly reduced, by approximately 1/5, in the non-ethanol-producing strain (Δ*adhE* strain) (Figure 5), suggesting that the chain transfer reaction and alcoholysis reaction for ethanol were major factors involved in the production of 3HBOs. It was previously reported that 3HBO production was increased up to 24-fold by the addition of alcohol (DEG) [17]. Thus, an efficient 3HBO production system should be constructed by considering not only the amount of PHA synthase but also the alcohol type and concentration.

The association of two types of chaperones (GroEL-GroES and TF) and the expression level of PHA synthases (PhaR_YB4_ and PhaC_YB4_) were supported by data from the pET system-based assay (Figure 4). Previously, it was reported that the GroEL-GroES system acts mainly on 20–50 kDa proteins because of the steric limitation of its cylindrical cavity [33,39]. The sizes of the PhaR_YB4_ and PhaC_YB4_ proteins were 18.5 and 41.7 kDa, respectively which were within <50 kDa; however, overexpression of GroEL-GroES did not increase the expression level and solubility of PhaR_YB4_ and PhaC_YB4_. By comparison, coexpression of the two chaperone systems GroEL-GroES and TF increased the solubility of PhaR_YB4_ and PhaC_YB4_ 3.0- and 8.5-fold, respectively, suggesting that TF was the key chaperone that assists in the folding of active PhaR_YB4_ and PhaC_YB4_. Similarly, the effect of chaperones on the expression of a class I PHA synthase from *R. eutropha* H16 (PhaC_Re_) was confirmed in a previous study [29]. Comparison of the soluble and insoluble fractions of PhaC_Re_ showed that coexpression of two chaperone systems (GroEL-GroES and TF) enhanced the solubilization of PhaC_Re_ compared with that of one chaperone system (GroEL-GroES). The same tendency has been observed in other studies on reconstituted cell-free translation systems of *E. coli* proteins [39,40]. In the cell-free system, it was observed that TF had only a modest effect on the reduction in protein aggregation by itself but could prevent the aggregation of recalcitrant *E. coli* proteins by cooperating with other chaperone systems, including GroEL-GroES [39]. Therefore, it could be considered that the increased solubilization (expression level) of PhaR_YB4_ and PhaC_YB4_ was caused by overexpression of two chaperone systems (GroEL-GroES and TF). Additional cultivation of *E. coli* BW25113/pGEM-*phaRC_YB4_AB* + pG-KJE8 (GroEL-GroES expressing-strain) also supported this hypothesis since 3HBO productivity and PHA synthase activity were not changed from those of the chaperone nonexpressing strain (3HBO production: Control, 0.92 ± 0.05 g/L; +pG-KJE8, 0.73 ± 0.09 g/L, PhaRC_YB4_ activity: Control, 0.12 ± 0.06 U/mg-protein; +pG-KJE8, 0.18 ± 0.06 U/mg-protein).

## 5. Conclusions

This study demonstrated that the coexpression of chaperone proteins is an effective method for increasing the amount of the active form of PhaRC_YB4_ and the yield of 3HBOs in *E. coli* strains. Exploration of more suitable chaperone systems for achieving the best expression balance for related enzymes, such as PHA synthase, PhaA, and PhaB, may be a useful strategy for improving the efficiency of 3HBO production.

## Figures and Tables

**Table 1:** Strains and plasmids used in this study.

Strain or Plasmid	Relevant Characteristics	Reference/Source
Strains		
*Escherichia coli* BW25113	*rrnB*T14 Δ*lac*ZWJ16 *hsdR*514 Δ*araBAD*AH33	[35]
*E. coli* BW25113 Δ*adhE*	BW25113, Δ*adhE*::*FRT*-*Km*-*FRT*	[35]
*E. coli* BL21(DE3)	F^−^ *ompT hsdS*_B_(r_B_^−^m_B_^−^) *gal dcm*	Novagen
Plasmids		
pGEM-*phaRC*_YB4_*AB*	pGEM-T derivative; *pha*_Re_ promoter, *phaRC*_YB4_ from *B. cereus* YB-4, and *phaAB*_Re_ from *Ralstonia eutropha* (*Cupriavidus necator*), Ap^r^	[34]
pG-Tf2	Expression vector for GroEL/GroES/Tf; *pzt1* promoter; Cm^r^	Takara Bio.
pG-KJE8	Expression vector for GroEL/GroES with *pzt1* promoter; DnaK, DnaJ, GrpE with *araB* promoter; Cm^r^	Takara Bio.
pET15b-*phaR*_YB4_	pET15b derivative; expression of N-terminal His-tagged *phaR*_YB4_ from *B. cereus* YB-4, Ap^r^	[25]
pET15b-*phaC*_YB4_	pET15b derivative; expression of N-terminal His-tagged *phaC*_YB4_ from *B. cereus* YB-4, Ap^r^	[25]

**Table 2:** Effect of coexpression of the chaperone plasmid pG-Tf2 on 3HBO production in recombinant *E. coli* BW2511 harboring pGEM-*phaRC*_YB4_*AB.*

Plasmid	Dry Cell Weight (g/L)	Residual Cell Mass (g/L)	Intracellular P(3HB) (g/L)	Intracellular 3HBOs (g/L)	Extracellular 3HBOs (g/L)	Extracellular 3HB (g/L)	Molecular Weight of P(3HB)		
							*M_n_* (×10^4^)	*M_w_* (×10^4^)	*M_w_*/*M_n_*
pGEM-*phaRC*_YB4_*AB*	9.40 ± 0.05	1.72 ± 0.01	7.41 ± 0.09	0.27 ± 0.03	0.65 ± 0.04	0.03 ± 0.00	2.3 ± 0.0	5.0 ± 0.1	2.2
pGEM-*phaRC*_YB4_*AB* + pG-Tf2	8.81 ± 0.08	2.22 ± 0.12	6.08 ± 0.15	0.50 ± 0.04	1.24 ± 0.08	0.03 ± 0.00	1.7 ± 0.1	3.5 ± 0.0	2.2

Cells were cultivated in LB medium containing 20 g/L glucose and the appropriate antibiotics at 30 °C for 48 h. The amount and molecular weight of P(3HB) were determined by GC and GPC analyses, respectively. The concentrations of intracellular/extracellular 3HBOs and 3HB were measured by an enzyme assay. The results are the averages ± standard errors from three independent experiments.

**Table 3:** Effect of coexpression of the chaperone plasmid pG-Tf2 on 3HBO production in recombinant *E. coli* BW2511Δ*adhE* harboring pGEM-*phaRC*_YB4_*AB*.

Plasmid	Dry Cell Weight (g/L)	Residual Cell Mass (g/L)	Intracellular P(3HB) (g/L)	Intracellular 3HBOs (g/L)	Extracellular 3HBOs (g/L)	Extracellular 3HB (g/L)	Molecular Weight of P(3HB)		
							*M_n_* (×10^4^)	*M_w_* (×10^4^)	*M_w_* /*M_n_*
pGEM-*phaRC*_YB4_*AB*	9.67 ± 0.02	1.42 ± 0.09	8.24 ± 0.07	0.01 ± 0.00	0.18 ± 0.01	0.04 ± 0.00	20.4 ± 1.3	151.9 ± 5.4	7.5
pGEM-*phaRC*_YB4_*AB* + pG-Tf2	9.77 ± 0.02	2.03 ± 0.23	7.73 ± 0.21	0.02 ± 0.00	0.37 ± 0.02	0.03 ± 0.00	9.4 ± 0.5	76.5 ± 5.8	8.2

Cells were cultivated in LB medium containing 20 g/L glucose and the appropriate antibiotics at 30 °C for 48 h. The amount and molecular weight of P(3HB) were determined by GC and GPC analyses, respectively. The concentrations of intracellular/extracellular 3HBOs and 3HB were measured by an enzyme assay. The results are the averages ± standard errors from three independent experiments.

## Data Availability

Not applicable.

## Supplementary Materials

The following supporting information can be downloaded at https://www.mdpi.com/article/10.3390/microorganisms10020458/s1, Figure S1: Molecular weight distribution of P(3HB) extracted from recombinant *E. coli* BW25113 WT (A) or Δ*adhE* (B) at 48 h of cultivation; Figure S2: Effect of the chaperone plasmid pG-Tf2 on the solubilization of PhaR_YB4_ and PhaC_YB4_; Table S1: Effect of coexpression of chaperones on 3HBO production in recombinant *E. coli* BW2511Δ*adhE* with various ethanol concentrations.
