# Supplementary material for: Enhanced Production of (R)-3-Hydroxybutyrate Oligomers by Coexpression of Molecular Chaperones in Recombinant Escherichia coli Harboring a Polyhydroxyalkanoate Synthase Derived from Bacillus cereus YB-4
Source: Microorganisms. 2022 Feb 16;10(2):458. doi: 10.3390/microorganisms10020458 (PMC8878867; doi:10.3390/microorganisms10020458)
Supplement: Supplementary file 1 [file microorganisms-10-00458-s001.zip › microorganisms-1593690-SI.pdf]

Supplementary Materials

Table S1. Effect of coexpression of chaperones on 3HBO production in recombinant *E. coli* BW2511*ΔadhE* with various ethanol concentrations. *E. coli* BW25113*ΔadhE* harboring pGEM-*phaRC*$_{YB4}$*AB* or pGEM-*phaRC*$_{YB4}$*AB*+pG-Tf2 were cultivated in LB medium containing 20 g/L glucose at 30 °C for 48 h with various ethanol concentration.

Ethanol [a] (g/L)	Plasmid	Dry cell weight (g/L)	Extracellular 3HBO (g/L)	Extracellular 3HB (g/L)	Ethanol at 48 h [b] (g/L)
0	pGEM-*phaRC*$_{YB4}$*AB*	9.65	0.10	0.03	Trace
	pGEM-*phaRC*$_{YB4}$*AB*+pG-Tf2	9.76	0.39	0.03	Trace
0.5	pGEM-*phaRC*$_{YB4}$*AB*	9.70	0.21	0.03	0.20
	pGEM-*phaRC*$_{YB4}$*AB*+pG-Tf2	9.21	0.62	0.03	0.30
1	pGEM-*phaRC*$_{YB4}$*AB*	9.36	0.20	0.04	0.58
	pGEM-*phaRC*$_{YB4}$*AB*+pG-Tf2	9.12	0.85	0.04	0.63
3	pGEM-*phaRC*$_{YB4}$*AB*	8.92	0.65	0.04	2.23
	pGEM-*phaRC*$_{YB4}$*AB*+pG-Tf2	8.50	1.35	0.04	2.30
5	pGEM-*phaRC*$_{YB4}$*AB*	8.48	1.23	0.03	4.05
	pGEM-*phaRC*$_{YB4}$*AB*+pG-Tf2	8.22	1.60	0.05	3.82
10	pGEM-*phaRC*$_{YB4}$*AB*	7.53	1.90	0.04	8.38
	pGEM-*phaRC*$_{YB4}$*AB*+pG-Tf2	7.56	2.07	0.07	8.02

[a] Ethanol was added at the beginning of cultivation. [b] Ethanol concentration in the culture supernatant after 48 h of cultivation.

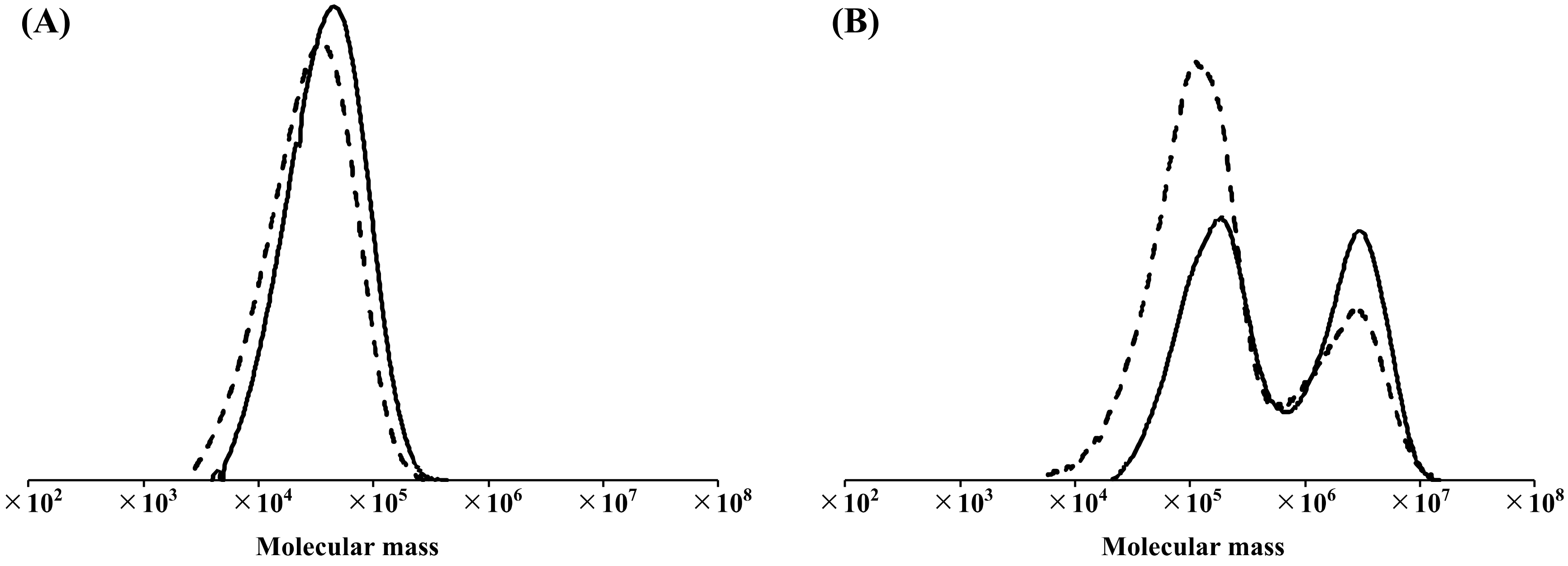

Figure S1. Molecular weight distribution of P(3HB) extracted from recombinant *E. coli* BW25113 WT (**A**) or *ΔadhE* (**B**) at 48 h of cultivation. Solid line, pGEM-*phaRC*$_{\mathrm{YB4}}$*AB*; broken line, pGEM-*phaRC*$_{\mathrm{YB4}}$*AB*+pG-Tf2.

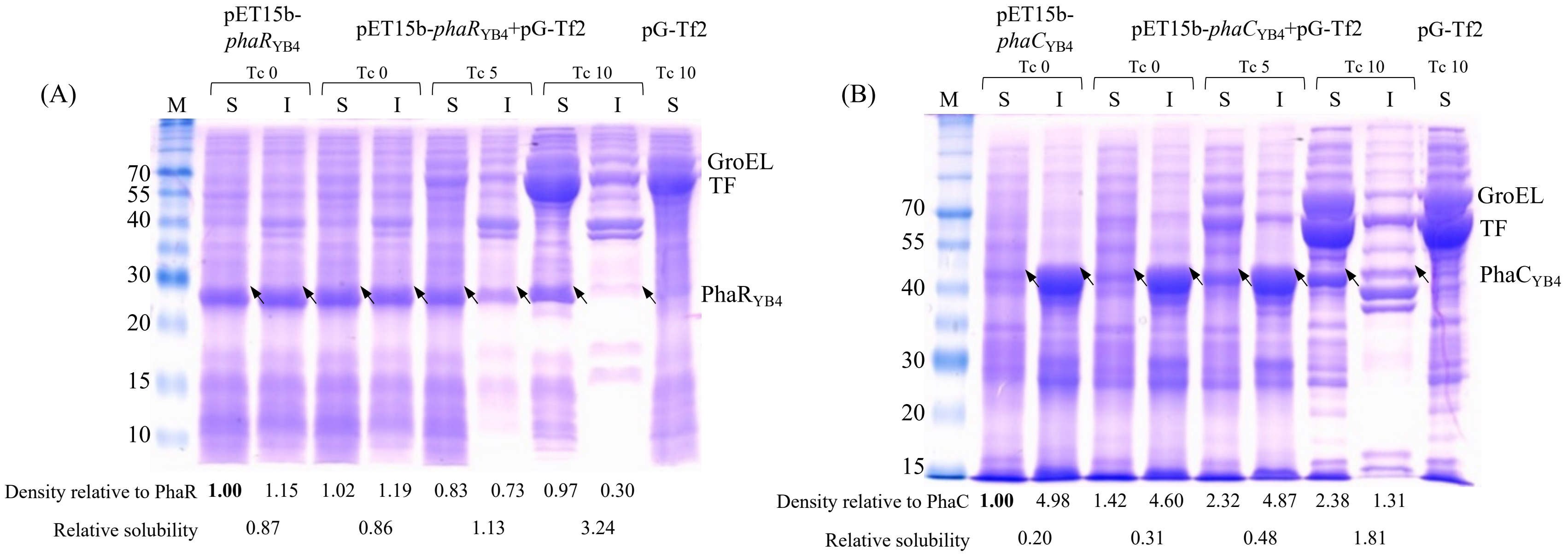

Figure S2. Effect of the chaperone plasmid pG-Tf2 on the solubilization of PhaR_{YB4} and PhaC_{YB4}. The levels of solubilized PhaR_{YB4} and PhaC_{YB4} were evaluated by SDS-PAGE. (**A**) *E. coli* BL21(DE3) harboring pET15b-*phaR*$_{\text{YB4}}$, pET15b-*phaR*$_{\text{YB4}}$+pG-Tf2 or pG-Tf2 and (**B**) *E. coli* BL21(DE3) harboring pET15b-*phaC*$_{\text{YB4}}$, pET15b-*phaC*$_{\text{YB4}}$+pG-Tf2 or pG-Tf2 were cultivated with 0-10 μg/L tetracycline (Tc). After sonication of each cell sample, soluble (S) and insoluble (I) fractions were analyzed by SDS-PAGE. The arrows indicate the bands corresponding to PhaR_{YB4} and PhaC_{YB4}. The relative densities were calculated using ImageJ software. The density relative to the band of PhaR_{YB4} or PhaC_{YB4} is the ratio of the density of each band to that of the band for the soluble fraction of PhaR_{YB4} or PhaC_{YB4} alone (bold). The relative solubilities are the ratios of the S to I fractions within each sample.
